# Assessment of Large Language Models in Colorectal Cancer Multidisciplinary Tumor Board Decision-Making: A Retrospective Single-Center Comparison of Guideline-Integrated General-Purpose vs. Domain-Specialized Models

**DOI:** 10.3390/curroncol33060309

**Published:** 2026-05-26

**Authors:** Aydan Farzaliyeva, Mehmet Nezir Ramazanoglu, Arzu Oguz, Ozden Altundag, Zafer Akcali

**Affiliations:** 1 Division of Medical Oncology, Department of Internal Medicine, Faculty of Medicine, Baskent University, 06490 Ankara, Türkiye; 2 Department of Medical Informatics, Faculty of Medicine, Baskent University, 06490 Ankara, Türkiye

**Keywords:** colorectal neoplasms, artificial intelligence, large language models, multidisciplinary tumor board, decision support systems, clinical, gemini, MedGemma

## Abstract

Background: Large language models (LLMs) are emerging as clinical decision-support tools in oncology, yet their ability to generate reliable treatment recommendations in real-world multidisciplinary tumor board (MTB) settings remains uncertain, particularly for complex colorectal cancer (CRC). Methods: In this retrospective study, 300 consecutive adult CRC cases discussed at a tertiary MTB were evaluated. Standardized de-identified case summaries were independently submitted to Gemini 2.5 (general-purpose, guideline-integrated) and MedGemma 27B (domain-specialized; T = 0.0 and T = 1.0). Concordance with MTB decisions was assessed using weighted Cohen’s kappa (κ), accuracy, F1 score, and recall. Safety was adjudicated by blinded senior MTB members using a three-tier risk framework. Importantly, Gemini 2.5 was evaluated in a guideline-integrated setting, whereas MedGemma operated without external guideline retrieval, introducing a predefined asymmetry in knowledge augmentation. Results: Gemini 2.5 demonstrated substantial agreement (κ = 0.792, *p* < 0.001), the highest exact concordance (85.0%), and a clinical safety rate of 83.7%, significantly outperforming MedGemma (*p* < 0.001). MedGemma showed moderate agreement at T = 0.0 (κ = 0.566; accuracy = 70.3%) and modest improvement at T = 1.0 (κ = 0.610; accuracy = 73.5%; *p* = 0.038), with lower safety rates (62.3% and 62.7%; *p* < 0.001). Discordance predominantly occurred in clinically complex scenarios, including surgical/interventional planning, active surveillance, recurrent disease management, and patients with compromised performance status. Conclusions: A guideline-integrated general-purpose LLM demonstrated superior concordance and safety compared with a domain-specialized model operating without external retrieval, supporting its adjunctive use within MTBs while preserving expert clinical judgment.

## Simple Summary

Artificial intelligence systems are increasingly being explored to support cancer care. In colorectal cancer, treatment decisions are typically made by multidisciplinary tumor boards that bring together specialists from different fields. However, it remains unclear whether advanced language-based artificial intelligence models can reliably assist in these complex decisions. In this study, we compared two large language models, Gemini and MedGemma, with real-world tumor board decisions in 300 patients with colorectal cancer. A guideline-integrated general-purpose model showed a high level of agreement with expert tumor board decisions and produced safer recommendations than a domain-specialized model operating without external knowledge integration. Differences were more frequent in clinically complex situations, such as surgical planning and recurrent disease. These findings suggest that well-designed artificial intelligence tools may complement multidisciplinary decision-making while preserving the central role of experienced clinicians.

## 1. Introduction

Multidisciplinary tumor boards (MTBs) were established to collaborative decision-making among specialists in medical oncology, surgery, radiology, pathology, and related disciplines. Although MTBs play a central role in modern oncology practice, they are resource-intensive and time-consuming, requiring the integration and interpretation of large volumes of heterogeneous clinical data. This challenge is particularly evident in colorectal cancer (CRC), where rising global incidence and increasingly complex management strategies have led to a growing number of cases discussed at MTBs, placing additional demands on healthcare resources [1].

In this context, artificial intelligence (AI) has emerged as a promising tool to support clinical decision-making. Recently, large language models (LLMs) have demonstrated potential to assist multidisciplinary tumor boards (MTBs) in various cancers, including gastric, breast, head and neck cancer, and sarcomas [2,3,4,5]. These models can help by rapidly summarizing and analyzing complex clinical information, synthesizing evidence, and providing decision support, as supported by several studies highlighting their utility as adjuncts in clinical decision-making [2,3,4,5]. AI applications have demonstrated value across multiple stages of CRC management, including screening, endoscopy, radiological and histopathological assessment, and surgical planning. However, studies exploring the potential of AI as an adjunctive tool in colorectal cancer multidisciplinary tumor boards are limited, with small sample sizes and fewer comprehensive evaluations [6,7].

Most general-purpose LLMs, while highly capable across a wide range of tasks, are not specifically optimized for medical applications and are typically deployed in cloud-based environments, where clinical data are processed on external servers outside direct institutional control.

To address these limitations, Google Research and Google DeepMind introduced MedGemma in July 2025, a suite of open-source, medically tuned foundation models designed specifically for the medical domain [8]. Unlike general-purpose LLMs such as Gemini 2.5, which is a large-scale, general-purpose model capable of incorporating external clinical guidelines through in-context learning, MedGemma is domain-specialized and can be locally deployed within institutional infrastructures. This enables clinical data to remain on-premises under full institutional governance, an important consideration for healthcare systems with strict data privacy and regulatory requirements.

In this retrospective study, we evaluated the performance of LLMs in CRC decision-making by comparing treatment recommendations generated by an MTB, considered the gold standard, with those produced by Gemini 2.5, a general-purpose, guideline-aware model, and MedGemma 27B, a domain-specialized medical language model optimized for text-only inputs. Accordingly, this study was designed with three predefined objectives: (i) to assess the concordance between LLM-generated recommendations and real-world MTB decisions; (ii) to evaluate the clinical safety of model outputs using a structured expert adjudication framework; and (iii) to examine the impact of model configuration, including temperature settings, on performance and recommendation patterns. Importantly, this comparison reflects a guideline-integrated general-purpose model versus a domain-specialized model operating without external retrieval.

## 2. Materials and Methods

### 2.1. Study Design and Patient Cohort

This retrospective observational study evaluated the concordance and clinical safety of treatment recommendations generated by LLMs compared with decisions made by MTB in patients with colorectal cancer.

Included in this study were 300 adult patients (≥18 years) diagnosed with colorectal cancer whose cases were presented at formal multidisciplinary tumor board meetings between January 2022 and December 2025. All patients were diagnosed and managed at a tertiary academic cancer center. The MTB decisions, made as part of routine clinical practice, served as the reference standard for treatment recommendations. For each patient, a comprehensive clinical case package was compiled, mirroring the information routinely presented during MTB meetings. Multidisciplinary tumor board treatment recommendations were classified into six predefined categories: surgery/interventional procedures, neoadjuvant or adjuvant chemotherapy, palliative chemotherapy, neoadjuvant or adjuvant chemoradiotherapy, active surveillance, and additional diagnostic or molecular testing. Patients were evaluated across different disease settings, including adjuvant, neoadjuvant, metastatic, and recurrent disease contexts. No additional exclusion criteria were applied beyond the availability of sufficient clinical information to allow the generation of treatment recommendations by the LLMs. The same standardized clinical information was provided to two large language models—Gemini 2.5 and MedGemma 27B—to generate independent treatment recommendations for each case. Model-generated recommendations were subsequently compared with the original MTB decisions to assess concordance and clinical safety.

**Figure 1:**
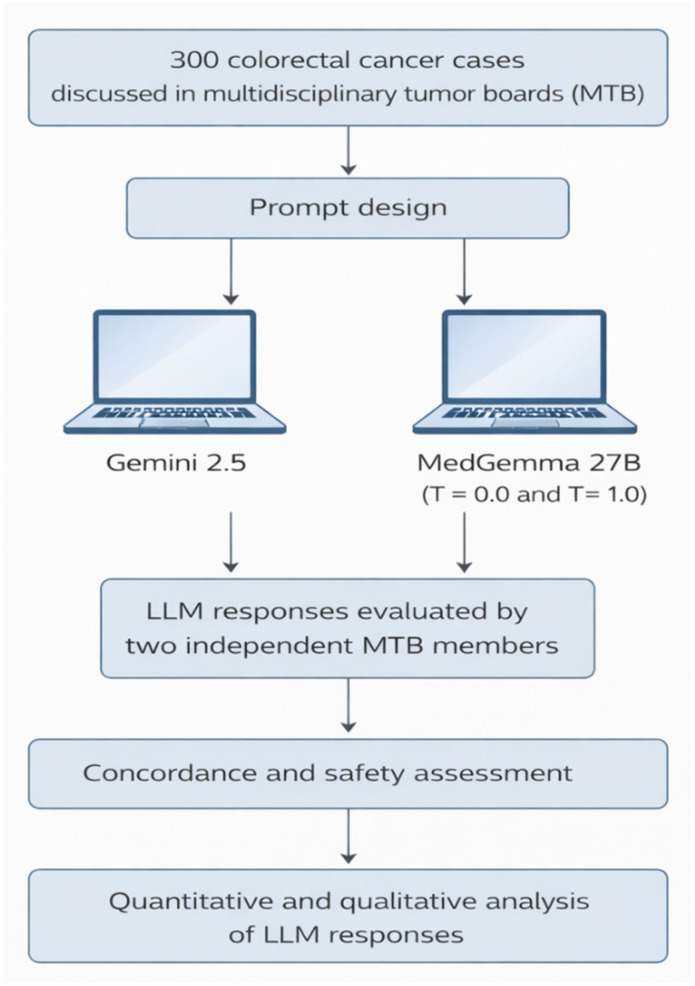
Study workflow and evaluation framework.

The study workflow, from case selection and prompt design to LLM evaluation and concordance analysis, is summarized in Figure 1.

### 2.2. Ethical Considerations

This study was approved by the Institutional Review Board (Project No: KA25/422) on 25 November 2025. The study was conducted in accordance with the principles of the Declaration of Helsinki and applicable ethical regulations. All patient data used in this study consisted of retrospectively collected clinical information. To protect patient privacy, no personally identifiable information was included or shared, and all data were anonymized prior to analysis. Patient confidentiality was strictly maintained throughout the study, and no data allowing direct or indirect patient identification were accessible to the large language models.

Medical texts were manually de-identified to meet the standards of both the HIPAA Privacy Rule and Turkey’s Personal Data Protection Law (KVKK). The 18 patient identifiers specified by HIPAA [9] were anonymized using methods from the official KVKK guide (Table 3.1) [10]: seventeen were removed via the ‘removal of variables’ technique, while dates were addressed with the ‘noise addition’ method, entailing their complete removal or fabrication.

### 2.3. LLM Input and Prompting Strategy

The MedGemma 27B model was downloaded from https://huggingface.co/google/medgemma-27b-text-it (accessed on 1 January 2026) and deployed on a local workstation equipped with four NVIDIA RTX 3090 GPUs (24 GB of VRAM each), providing a total of 96 GB of GPU memory. To support large-scale inference without relying on external cloud services, the model was served using vLLM v0.14.1 on Linux Mint 21.3.

In contrast, Gemini 2.5 was accessed through Google’s cloud-based inference infrastructure, with computational resources fully managed by the provider and no requirement for local hardware or GPU acceleration.

Gemini 2.5 was accessed via a cloud-based interface, and all queries were conducted between December 2025 and January 2026. As cloud-based models may be updated over time, the access period was recorded to enhance transparency and reproducibility.

To ensure alignment between AI-generated recommendations and evidence-based clinical practice, the NCI colorectal cancer treatment guidelines were integrated as in-context reference material for the Gemini 2.5 model [11,12]. Leveraging the architecture’s extensive context window capacity, the guideline content was extracted in HTML format and converted into a structured JSON representation. This conversion preserved the essential hierarchical organization of sections, tables, and treatment algorithms, thereby facilitating precise parsing and contextual comprehension by the LLM while mitigating the information loss frequently associated with unstructured formats, such as PDF or plain text.

Methodologically, this approach utilized long-context prompting rather than traditional Retrieval-Augmented Generation (RAG). By presenting the comprehensive, structured NCI guidelines directly within the prompt context, the model maintained immediate access to the full clinical dataset without the need for external document retrieval or vector database indexing.

For the Gemini 2.5 evaluation, the model input was structured as a singular, comprehensive prompt containing a standardized system prompt (~500 tokens), the JSON-formatted NCI colorectal cancer guidelines (~85,000 tokens), and patient-specific clinical data (~3200 tokens per case). These inputs were processed within a single, contiguous context window without truncation, leveraging the model’s architectural capacity for Long-Context In-Context Learning (LC-ICL).

In contrast, MedGemma 27B—a domain-specialized LLM extensively pre-trained on biomedical and clinical corpora—was evaluated without external guideline augmentation. The input for MedGemma 27B was limited to the standardized system prompt (~500 tokens) and the patient-specific clinical information (~3200 tokens per case), both of which remained well within the model’s native context window. Consequently, MedGemma 27B generated treatment recommendations derived exclusively from its internally encoded medical knowledge and specialized weights, serving as a baseline for specialized pre-training versus real-time guideline integration.

Gemini 2.5 is a general-purpose model not specifically trained for medical use, and in our study its performance was adapted to the clinical context through structured guideline integration using long-context prompting. In contrast, MedGemma 27B is described by its developers as a domain-specialized model that has been trained and fine-tuned on medical data; therefore, we did not apply additional long-context guideline prompting to this model, as it was intended to operate based on its internally learned domain-specific knowledge rather than externally injected guideline content.

Due to infrastructure and context-window limitations associated with our local MedGemma 27B deployment, incorporating an additional arm with equivalent large-scale guideline input (~85k tokens) was not feasible. We have explicitly addressed this input asymmetry in the manuscript by clarifying the differences in guideline integration between the two models.

A standardized expert-level prompt was applied consistently across both models, instructing them to assume the role of an expert physician participating in a multidisciplinary tumor board and to generate evidence-based treatment recommendations. Model-specific prompt formulations are provided in Supplementary Tables S1 and S2.

All prompts were finalized before the analysis and were not iteratively modified using the study cases. The same standardized prompt structure was applied consistently across all cases and models.

Each patient case was presented using a uniform clinical input format that mirrored real-world tumor board presentations, and each case was evaluated in a separate, newly initiated session to prevent contextual carryover between cases. Within the prompt, the models were instructed to prioritize outputs as a primary recommendation, an alternative recommendation, and suggested further investigations, reflecting routine multidisciplinary decision-making processes.

Gemini 2.5 was evaluated using a fixed temperature setting of 1.0, which is the default for this model. For MedGemma 27B, two temperature settings (T = 0.0 and T = 1.0) were assessed to examine the impact of response variability on concordance and clinical safety. Although the MedGemma technical report recommends a temperature of 0.0 [8], we deemed it necessary to provide empirical evidence for this setting. Finally, model outputs were restricted to treatment recommendations and subsequently mapped to the predefined six-category treatment classification framework.

### 2.4. Concordance and Safety Assessment

Large language model–generated treatment recommendations underwent a formal, predefined, and blinded adjudication process to assess both concordance with MTB decisions and clinical safety. Evaluations were performed independently by two independent senior members of the MTB, each blinded to the other’s assessment and to the identity of the LLM producing the recommendation, thereby minimizing observer and attribution bias.

Concordance between LLM-generated recommendations and the reference MTB decisions was evaluated using a three-tier ordinal classification framework:

- Fully concordant, defined as complete alignment with the MTB decision regarding treatment modality and overall clinical intent;
- Partially concordant, defined as recommendations that were clinically acceptable but demonstrated deviations in therapeutic sequencing, treatment intensity, or scope relative to the MTB decision;
- Discordant, defined as recommendations that were incongruent with the MTB decision or considered clinically inappropriate within the given clinical context.

Clinical safety was assessed independently using a predefined three-level risk stratification schema:

- Safe, indicating recommendations deemed appropriate and unlikely to result in adverse clinical consequences;
- Potentially risky, indicating recommendations that could lead to suboptimal management, delayed initiation of appropriate therapy, or necessitate heightened clinical caution;
- Harmful, indicating recommendations with a plausible risk of causing clinically meaningful patient harm if implemented.

In instances of inter-rater disagreement, cases were subjected to structured joint re-evaluation, during which discrepancies were resolved through discussion until a consensus determination was reached. This consensus classification constituted the final adjudicated outcome and was used for all subsequent concordance and safety analyses.

### 2.5. Statistical Analysis

Continuous variables are presented as mean ± standard deviation or median (minimum–maximum), and categorical variables as counts (*n*) and percentages (%). Concordance between large language model recommendations and MTB decisions was assessed using the weighted Cohen’s kappa (κ) coefficient for multicategorical nominal data, with interpretation based on the Landis and Koch classification [13]. Model performance was further evaluated using accuracy, exact agreement (%), F1 score, and recall. Accuracy was defined as the proportion of cases in which the treatment recommendation generated by the large language model exactly matched MTB decision among all evaluated cases. Recall was calculated as the proportion of MTB treatment decisions correctly identified by the model within each treatment category, reflecting the model’s ability to capture appropriate clinical decisions without omission. The F1 score, representing the harmonic mean of precision and recall, was used to provide a balanced measure of model performance by accounting for both false-positive and false-negative recommendations, particularly in the presence of class imbalance across treatment categories. Confidence intervals were not reported due to the multicategorical structure of the analyses and instability of estimates in low-frequency cells.

Comparisons of concordance (full/partial/discordant) and clinical safety (safe/potentially risky/harmful) distributions across different large language models within the same patient cohort were performed using the Marginal Homogeneity test for paired multicategorical data. Pairwise model comparisons (Gemini 2.5 vs. MedGemma T = 0.0, Gemini 2.5 vs. MedGemma T = 1.0, and MedGemma T = 0.0 vs. T = 1.0) were conducted separately using this test.

The distributions of concordance and clinical safety levels across demographic and clinical variables were analyzed using the chi-square test. When significant differences were detected, Bonferroni-adjusted post hoc analyses were applied to control for the inflation of type I error due to multiple comparisons. Specifically, pairwise comparisons across the demographic and clinical subgroups were performed, with *p*-values adjusted using the Bonferroni correction. This method ensures that the overall family-wise error rate remains controlled despite the large number of subgroup tests. A *p* value < 0.05 was considered statistically significant for all analyses. Statistical analyses were performed using IBM SPSS Statistics version 31.0 (IBM Corp., Armonk, NY, USA).

## 3. Results

A total of 300 colorectal cancer patients discussed at multidisciplinary tumor boards (MTBs) were included in the study. The clinical and demographic characteristics of the study population are presented in detail in Table 1.

The median age at diagnosis was 69 years (range, 25–97), and 52.7% of patients were male. Most patients were aged ≥65 years (71.3%). According to TNM staging, 10.7% of patients had stage I, 33.0% stage II, 39.0% stage III, and 17.3% stage IV disease. According to the Charlson Comorbidity Index, 57.0% of patients had a mild-to-moderate comorbidity burden (score 1–2), whereas 16.3% had a high comorbidity burden (score ≥ 3). ECOG performance status was 0–1 in 81.3% of patients. Tumors were located in the colon in 78.7% and in the rectum in 21.3% of patients. Microsatellite instability status was microsatellite stable in 64.7% of cases, MSI-unstable in 7.3%, and not available in 28.0%. Most patients were discussed in the adjuvant disease setting (72.3%).

Concordance Between Multidisciplinary Tumor Board Decisions and AI-Generated Treatment Recommendations

The concordance of Gemini 2.5, MedGemma (T = 0.0), and MedGemma (T = 1.0) models with MTB decisions is presented in Table 2 and Table 3, including treatment group–specific distributions, weighted Cohen’s kappa coefficients, accuracy, F1 scores, and recall values.

The Gemini 2.5 model demonstrated a high level of agreement with MTB treatment decisions, with a weighted Cohen’s kappa of 0.792 (*p* < 0.001). An accuracy (exact agreement) rate of 85.0% indicated that Gemini recommended the identical treatment category as the MTB in the majority of cases. In addition, the F1 score (0.79) and Recall (0.82) reflected balanced performance across treatment modalities, with a low likelihood of missing appropriate MTB decisions. Agreement was particularly high for palliative chemotherapy (92.9%), additional radiological and molecular testing (90.0%), and active surveillance (89.3%) decisions, whereas human expert judgment remained essential for multimodal treatment strategies, particularly surgical/interventional procedures and chemoradiotherapy. Agreement between MedGemma models (T = 0.0 and T = 1.0) and MTB treatment decisions was further analyzed across treatment categories. MedGemma T = 0.0 demonstrated the highest exact concordance for adjuvant–neoadjuvant chemotherapy (92.9%), additional molecular/radiological testing (85.0%), and palliative chemotherapy (71.4%). Similarly, MedGemma T = 1.0 achieved its highest concordance rates in adjuvant–neoadjuvant chemotherapy (92.1%), additional molecular/radiological testing (90.0%), and neoadjuvant/adjuvant chemoradiotherapy (75.8%).

The lowest concordance rates for both temperature settings were observed in active surveillance (39.3% for T = 0.0 and 44.0% for T = 1.0) and surgical/interventional procedures (11.1% and 22.2%, respectively). Overall, MedGemma T = 1.0 showed improved agreement with MTB decisions compared with T = 0.0.

**Figure 2:**
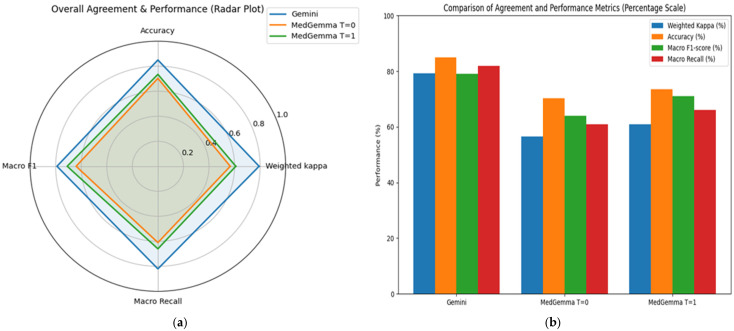
(**a**) Radar plot comparing the overall concordance and performance of the Gemini and MedGemma large language models with multidisciplinary tumor board decisions based on weighted Cohen’s kappa, accuracy, F1 score, and recall; (**b**) Bar chart comparison of concordance and performance metrics (weighted Cohen’s kappa, accuracy, macro F1 score, and macro recall) between Gemini and MedGemma (T = 0.0 and T = 1.0) large language models and multidisciplinary tumor board decisions.

In terms of global agreement metrics, MedGemma T = 0.0 demonstrated moderate agreement with MTB decisions, with a weighted Cohen’s kappa of κ = 0.566 (*p* < 0.001), whereas MedGemma T = 1.0 achieved good agreement (κ = 0.610, *p* < 0.001). Accuracy rates increased from 70.3% (T = 0.0) to 73.5% (T = 1.0), indicating a higher proportion of exact concordance with MTB decisions at the higher temperature setting. Consistently, improvements in F1 score (0.64 vs. 0.71) and recall (0.61 vs. 0.66) suggest that the T = 1.0 setting provided more balanced classification performance across treatment groups and reduced the likelihood of missing appropriate MTB recommendations. The comparative performance profiles of the evaluated models are visually summarized in Figure 2.

### Comparative Analysis of Concordance and Safety Between Gemini and MedGemma Large Language Models

AI-generated recommendations, including detailed chemotherapy and radiotherapy regimens, and other treatment modalities, were independently reviewed by two senior MTB members for concordance with MTB decisions and clinical safety. Concordance was assessed using a structured three-tier concordance framework, with recommendations classified as fully concordant, partially concordant, or discordant. Concurrently, clinical safety was evaluated using a predefined three-category safety framework: safe, potentially risky, and harmful.

**Figure 3:**
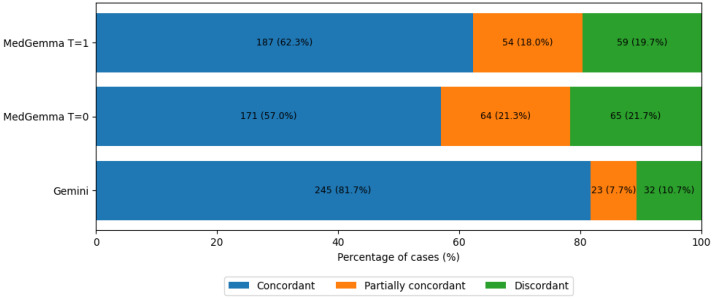
Concordance of treatment recommendations generated by Gemini 2.5 and MedGemma (T = 0.0 and T = 1.0) with MTB decisions.

**Figure 4:**
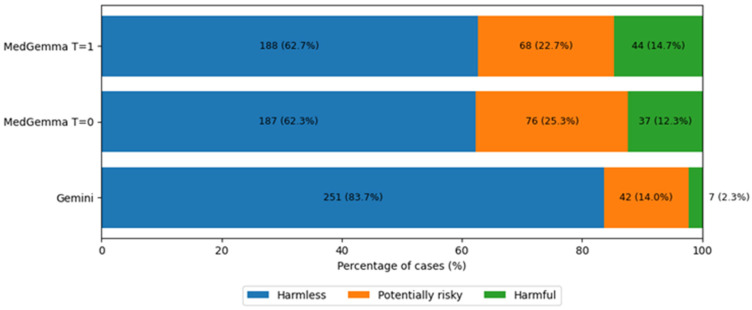
Safety assessment of treatment recommendations generated by Gemini 2.5 and MedGemma (T = 0.0 and T = 1.0).

The concordance distributions of the Gemini 2.5, MedGemma (T = 0.0), and MedGemma (T = 1.0) AI models with MTB decisions are shown in Figure 3, while the safety outcomes are shown in Figure 4.

In terms of concordance, Gemini 2.5 demonstrated the highest full concordance rate with MTB decisions (81.7%), compared with 57.0% for MedGemma (T = 0.0) and 62.3% for MedGemma (T = 1.0). Comparisons of the three-category concordance distributions showed significant differences between Gemini and MedGemma (T = 0.0) (*p* < 0.001) and between Gemini and MedGemma (T = 1.0) (*p* < 0.001). Additionally, MedGemma (T = 1.0) showed a statistically significant improvement in concordance compared with MedGemma (T = 0.0) (*p* = 0.038).

In the safety assessment, Gemini 2.5 demonstrated the highest proportion of safe recommendations (83.7%), whereas MedGemma (T = 0.0: 62.3% and T = 1.0: 62.7%) showed higher rates of potentially risky and harmful recommendations. Safety distribution comparisons revealed significant differences between Gemini 2.5 and MedGemma (T = 0.0) (*p* < 0.001) and between Gemini 2.5 and MedGemma (T = 1.0) (*p* < 0.001), while no significant difference was observed between MedGemma (T = 0.0) and MedGemma (T = 1.0) (*p* = 0.567). These findings indicate that although increasing the temperature improved MedGemma’s concordance, it did not result in a meaningful improvement in its safety profile.

Differences in concordance and safety between the Gemini 2.5, MedGemma (T = 0.0), and MedGemma (T = 1.0) AI models relative to multidisciplinary tumor board (MTB) decisions are presented in Table 4.

Comparative Analysis of Concordance Between Large Language Models and Multidisciplinary Tumor Board Decisions by Demographic and Clinical Variables.

The comparison of concordance levels between large language models and multidisciplinary tumor board decisions across demographic and clinical variables is presented in Table 5.

Across demographic and clinical variables, concordance between Gemini 2.5 and MTB decisions did not differ significantly by sex, age at diagnosis, comorbidity status, ECOG performance status, tumor location, or MSI status (all *p* > 0.05). In contrast, concordance levels differed significantly according to TNM stage, treatment setting, and MTB treatment modality. Discordance was more frequent among patients with stage IV disease, whereas fully and partially concordant decisions predominated in stages I–III (*p* = 0.033). With respect to treatment setting, discordance was markedly higher in recurrent cases, while concordance rates were more comparable across adjuvant, neoadjuvant, and metastatic settings (*p* < 0.001).

Significant variation in concordance was observed across MTB treatment modalities (*p* < 0.001). Discordance was more common for surgical/interventional procedures and adjuvant–neoadjuvant chemoradiotherapy, whereas Gemini showed higher full concordance for adjuvant–neoadjuvant chemotherapy, palliative chemotherapy, and additional radiological and molecular testing decisions.

At both temperature settings (T = 0.0 and T = 1.0), concordance between MedGemma and multidisciplinary tumor board (MTB) decisions did not differ significantly by sex, age at diagnosis, comorbidity classification, tumor localization, or primary tumor laterality (all *p* > 0.05), indicating consistent performance across these demographic and anatomical variables.

In contrast, concordance varied significantly according to TNM stage, ECOG performance status, MSI status, disease setting, and MTB treatment modality at both temperature settings. Higher rates of partial concordance and discordance were observed in stage II disease, whereas full concordance predominated in stage III (*p* < 0.001 for both T = 0.0 and T = 1.0). Discordance was more frequent in patients with ECOG ≥ 2 compared with those with ECOG 0–1 (T = 0.0: *p* = 0.023; T = 1.0: *p* = 0.036), and in MSI-unstable or MSI-unknown tumors compared with MSI-stable disease (*p* < 0.001 for both settings). Discordance was also higher in recurrent disease than in adjuvant, neoadjuvant, or metastatic settings (T = 0.0: *p* = 0.010; T = 1.0: *p* = 0.008). The greatest variation was observed across MTB treatment modalities, with higher discordance for surveillance and surgical/interventional decisions and higher concordance for adjuvant–neoadjuvant chemotherapy and chemoradiotherapy (*p* < 0.001 at both temperature settings).

The comparative analysis of clinical safety levels between the Gemini and MedGemma (T = 0.0 and T = 1.0) large language models and multidisciplinary tumor board decisions across demographic and clinical variables is presented in Table 6.

## 4. Discussion

Current artificial intelligence applications in colorectal cancer have predominantly focused on screening, diagnosis, prognostic stratification, and treatment response prediction, rather than direct treatment decision-making [6,7,14]. Although large language models have recently been proposed as tools to reduce oncologists’ workload and facilitate access to up-to-date clinical information, their role in generating or supporting multidisciplinary treatment recommendations in colorectal cancer remains largely unexplored. Accordingly, this study was designed to evaluate LLM performance in colorectal cancer decision-making by comparing model-generated recommendations with real-world MTB decisions, considered the clinical reference standard. Importantly, the comparison reflects two distinct approaches: a guideline-integrated general-purpose model and a domain-specialized model operating without external knowledge augmentation.

In this retrospective analysis of 300 colorectal cancer cases discussed at multidisciplinary tumor boards, a guideline-integrated general-purpose LLM, Gemini 2.5, demonstrated a high level of agreement with MTB treatment decisions, achieving substantial concordance (weighted Cohen’s κ = 0.792) and a high exact agreement rate (85.0%), whereas a domain-specialized model operating without external retrieval, MedGemma 27B, showed moderate concordance that improved modestly with higher temperature settings. In parallel, clinical safety assessment revealed a significantly higher proportion of safe recommendations for Gemini 2.5 (83.7%) compared with MedGemma, underscoring differences not only in agreement but also in patient safety profiles.

The highest rates of discordance for Gemini 2.5 were observed in Stage IV disease, surgical and interventional procedures, neoadjuvant and adjuvant chemoradiotherapy decisions, and recurrent disease underscoring the continued necessity of human expert clinical judgment in managing high-complexity oncological scenarios.

For MedGemma, the lowest concordance was observed in patients with ECOG performance status ≥ 2, stage II disease, MSI-high tumors, recurrent cases, and scenarios in which the MDT recommended active surveillance, surgery, or interventional procedures.

These subgroup findings should be interpreted with caution, particularly in smaller subgroups such as MSI-high and recurrent disease, where limited sample sizes increase the risk of type I error and over-interpretation. Accordingly, these analyses should be considered exploratory rather than confirmatory.

Prior to the emergence of LLMs, artificial intelligence–based treatment support in colorectal cancer was largely represented by guideline-driven clinical decision support systems (CDSSs), most notably IBM Watson for Oncology. In two independent clinical studies—one evaluating blinded expert agreement in a cohort of 99 patients, and another prospectively comparing Watson recommendations with multidisciplinary tumor board decisions in approximately 250 colorectal cancer patients—high concordance rates were reported, particularly in standardized stage II–III scenarios. However, concordance definitions were permissive, as multidisciplinary decisions were considered concordant if they appeared in either the “recommended” or the “for consideration” categories, thereby including secondary or alternative treatment options. Importantly, these systems relied on rule-based guideline mapping rather than contextual reasoning, underscoring that earlier AI-assisted approaches in colorectal cancer were designed to support guideline adherence rather than to emulate multidisciplinary treatment decision-making [15,16].

In an early experience study, Choo and colleagues evaluated the performance of a large language model (ChatGPT 4.0) by comparing model-generated management recommendations with multidisciplinary tumor board (MDT) decisions in a cohort of 30 patients with complex colorectal cancer. Overall concordance between ChatGPT and MDT decisions was reported to be high, with agreement rates of approximately 85–87% for overall treatment strategy, particularly with respect to surgical intent and the use of neoadjuvant or adjuvant therapies. However, the analysis focused on high-level management decisions, and detailed chemotherapy regimens, radiotherapy modalities, and nuanced multidisciplinary considerations were not assessed, underscoring the preliminary and exploratory nature of LLM use in colorectal cancer treatment planning [17].

Chatziisaak et al. evaluated the concordance between ChatGPT-4–generated treatment recommendations and multidisciplinary tumor board (MDT) decisions in a retrospective cohort of 100 patients with colorectal cancer. Complete concordance was observed in 72.5% of pretherapeutic MDT decisions and increased to 82.8% in post-therapeutic discussions, with partial concordance rates of 10.2% and 11.8%, respectively, indicating a high level of agreement at the level of overall treatment strategy, including surgical intent and the use of neoadjuvant or adjuvant therapy. However, detailed chemotherapy regimens and radiotherapy modalities were not assessed, and concordance was lower in patients aged ≥77 years and in those with high surgical risk (ASA ≥ III), highlighting the model’s limited ability to account for frailty, functional reserve, and overall comorbidity burden; notably, no Cohen’s kappa or other formal inter-method agreement statistics were reported, as concordance was evaluated using categorical agreement rates [18].

In a single-center pilot study by Nir Horesh, M.D., and colleagues, including 15 patients with colorectal and anal cancer, ChatGPT-generated management recommendations were compared with MDT decisions. Overall concordance between ChatGPT and MDT was rated high by expert reviewers (mean concordance score: 4.08), although agreement was assessed qualitatively rather than through direct model–MDT statistical metrics. Interrater agreement among reviewers assessing concordance was moderate, with Cohen’s κ values ranging from 0.333 to 0.577, whereas agreement regarding the justification of recommendations was low (κ range: 0.047–0.094), underscoring the exploratory, proof-of-concept nature of this pilot study [19].

With the recent release of MedGemma, the clinical evaluation of this model remains limited, with only a small number of published studies predominantly focusing on medication safety, information extraction, ophthalmology, or endoscopic image interpretation [20,21,22,23,24,25]. Notably, no prior studies have specifically examined the performance of MedGemma in medical oncology decision-making or multidisciplinary tumor board–level treatment recommendations.

In the only available multimodal clinical study evaluating cystoscopic image interpretation, general-purpose multimodal models achieved near-expert accuracy, whereas the domain-specific MedGemma-27B demonstrated comparatively lower diagnostic performance, particularly in challenging lesions such as carcinoma in situ and borderline tumors—entities that require nuanced, high-level clinical reasoning analogous to complex oncologic decision-making. These limitations were attributed to the absence of modality-specific pretraining data and constrained model capacity, which restricted reasoning depth and generalization in diagnostically complex scenarios [24].

Complementing these findings, Modi et al. investigated failures of clinical reasoning in large language models using oncology-based tasks—most notably AJCC cancer staging—as a stress test for high-level, multi-step inference. In that analysis, MedGemma exhibited markedly poor performance, attributed to limitations in hierarchical reasoning and insufficient separation of clinically distinct concepts despite extensive medical-domain pretraining [25]. Although this work focused on reasoning failures rather than treatment decision-making, it provides an important conceptual framework for interpreting our findings.

Extending this emerging evidence to real-world oncology practice, our study demonstrates that, despite being pretrained on biomedical text and medical imaging data, MedGemma shows lower concordance with multidisciplinary tumor board decisions in clinically complex scenarios. While its recommendations were often broadly aligned with established guideline principles, they frequently lacked adequate adaptation to patient-specific clinical context, particularly in situations requiring nuanced judgment rather than straightforward guideline application. These findings are consistent with prior evidence suggesting that reliance on narrative guideline content alone may be insufficient for safe and effective clinical decision support, particularly in complex oncologic scenarios such as oligometastatic or potentially curative-intent disease, where structured decision-making frameworks are critical. In this context, the integration of validated clinical scoring systems alongside guideline-based reasoning may improve both the robustness and clinical safety of AI-assisted recommendations [26,27].

An important methodological consideration in this study is the asymmetry in knowledge integration strategies between the two models. While identical clinical case inputs were provided, Gemini 2.5 was augmented through structured long-context guideline prompting, whereas MedGemma was evaluated based on its internally learned domain-specific knowledge without external guideline injection, consistent with its intended design. Therefore, the observed performance differences likely reflect not only model architecture but also differences in access to structured, up-to-date clinical knowledge. Accordingly, our findings should be interpreted as a comparison between guideline-integrated long-context prompting and domain-specialized modeling without external augmentation, rather than a definitive comparison of model types alone. Due to infrastructure and context-window limitations, applying equivalent large-scale guideline integration to MedGemma was not feasible, and thus a fully controlled comparison could not be performed.

Collectively, these findings suggest that performance differences may be driven by the combination of structured guideline integration and general reasoning capability, rather than model type alone. In this context, real-time access to structured clinical knowledge may play a critical role in improving decision alignment and safety.

Importantly, these findings do not directly establish differences in underlying reasoning mechanisms, and interpretations related to concepts such as “agentic reasoning,” “hierarchical decision-making,” or “dynamic integration of clinical factors” should be considered hypothesis-generating rather than conclusive.

In addition, our results suggest that domain-specific pretraining alone may be insufficient in the absence of structured and up-to-date clinical knowledge integration, although this conclusion should be interpreted cautiously given the non-equivalent comparison framework between models.

Importantly, these findings also highlight that safe clinical deployment of LLMs requires not only model capability but also appropriate governance frameworks, including controlled knowledge retrieval, context-aware decision-making, and auditability of model outputs.

Taken together, this study represents, to our knowledge, one of the most comprehensive evaluations of concordance between LLM-generated recommendations and multidisciplinary tumor board decisions in colorectal cancer.

Collectively, these findings provide a clinically relevant framework for understanding the current capabilities and limitations of LLM-assisted decision-making in colorectal cancer management, while underscoring the need for prospective and controlled studies to define their role in real-world clinical workflows.

This study has several limitations. First, its single-center, retrospective design limits the generalizability of the findings across different institutions and clinical settings, and external validation is required. Second, although prior lines of therapy, ECOG performance status, and the Charlson Comorbidity Index were incorporated as proxies for case complexity, other important determinants of MTB complexity (such as ASA class, detailed frailty measures, and surgical risk) were not consistently available due to the retrospective nature of the data. Third, the absence of an external control condition (e.g., guideline-based recommendations or independent clinician assessments) limits the contextual interpretation of the observed concordance. Fourth, safety and concordance assessments were performed by members of the same tumor board that generated the reference decisions, introducing potential confirmation bias despite blinded evaluation. In addition, inter-rater agreement prior to consensus was not systematically recorded; however, discrepant cases were resolved through structured discussion between the two raters to reach a final consensus. Finally, although identical clinical case inputs were provided to all models, a degree of input asymmetry existed in terms of guideline integration strategies (external long-context prompting in Gemini versus reliance on internally learned knowledge in MedGemma), which may have influenced model outputs and limited their direct comparability.

## 5. Conclusions

Guideline-integrated general-purpose LLMs, exemplified by Gemini 2.5, demonstrated higher concordance with multidisciplinary tumor board decisions compared with a domain-specialized model without external retrieval. These findings suggest that performance may be influenced not only by model characteristics but also by access to structured, up-to-date clinical knowledge. Accordingly, domain-specific pretraining alone may be insufficient without external knowledge integration, although this interpretation is limited by the non-equivalent comparison design. Overall, LLMs show potential as adjunctive decision-support tools, warranting further prospective evaluation.

## Figures and Tables

**Table 1:** Overview of the patient cohort.

Parameters	Group	*n* (%)
Sex	Female	142 (47.3)
	Male	158 (52.7)
Age, years	<65	86 (28.7)
	65–74	102 (34.0)
	≥75	112 (37.3)
TNM stage	I	32 (10.7)
	II	99 (33.0)
	III	117 (39.0)
	IV	52 (17.3)
Charlson Comorbidity Index	0	80 (26.7)
	<2	171 (57.0)
	≥3	49 (16.3)
ECOG performance status	0–1	244 (81.3)
	≥2	56 (18.7)
Tumor location	Colon	236 (78.7)
	Rectum	64 (21.3)
Microsatellite instability status	MSI stable	194 (64.7)
	MSI unstable	22 (7.3)
	Not available	84 (28.0)
Treatment setting	Adjuvant	217 (72.3)
	Neoadjuvant	26 (8.7)
	Metastatic	40 (13.3)
	Recurrent disease	17 (5.7)
Prior lines of therapy	0	237 (79)
	1	35 (11.7)
	≥2	28 (9.3)

**Abbreviations:** TNM, Tumor–Node–Metastasis; ECOG, Eastern Cooperative Oncology Group.

**Table 2:** Concordance between multidisciplinary tumor board decisions and treatment recommendations generated by large language models (Confusion matrices).

		Multidisciplinary Tumor Board Decision							
LLM	Treatment Modality	Surgery/IP	Neo/Adjuvant CT	Palliative CT	Neo/Adjuvant CRT	AS	Additional Tests	Kappa Value	*p* Value
Gemini 2.5	Surgery/IP	3 (33.3)	0 (0.0)	0 (0.0)	1 (3.0)	1 (1.2)	0 (0.0)	**0.792**	**<0.001**
	Neo/Adjuvant CT	0 (0.0)	108 (85.7)	2 (7.1)	5 (15.2)	8 (9.5)	1 (5.0)		
	Palliative CT	6 (66.7)	8 (6.3)	**26 (92.9)**	0 (0.0)	0 (0.0)	1 (5.0)		
	Neo/Adjuvant CRT	0 (0.0)	1 (0.8)	0 (0.0)	25 (75.8)	0 (0.0)	0 (0.0)		
	Active surveillance	0 (0.0)	8 (6.3)	0 (0.0)	2 (6.1)	**75 (89.3)**	0 (0.0)		
	Additional tests	0 (0.0)	1 (0.8)	0 (0.0)	0 (0.0)	0 (0.0)	**18 (90.0)**		
MedGemma T = 0.0	Surgery/IP	1 (11.1)	0 (0.0)	0 (0.0)	0 (0.0)	0 (0.0)	0 (0.0)	**0.566**	**<0.001**
	Neo/Adjuvant CT	0 (0.0)	**117 (92.9)**	6 (21.4)	10 (30.3)	49 (58.3)	3 (15.0)		
	Palliative CT	7 (77.8)	7 (5.6)	**20 (71.4)**	0 (0.0)	1 (1.2)	0 (0.0)		
	Neo/Adjuvant CRT	1 (11.1)	1 (0.8)	2 (7.1)	23 (69.7)	1 (1.2)	0 (0.0)		
	Active surveillance	0 (0.0)	1 (0.8)	0 (0.0)	0 (0.0)	33 (39.3)	0 (0.0)		
	Additional tests	0 (0.0)	0 (0.0)	0 (0.0)	0 (0.0)	0 (0.0)	**17 (85.0)**		
MedGemma T = 1.0	Surgery/IP	2 (22.2)	0 (0.0)	0 (0.0)	0 (0.0)	0 (0.0)	0 (0.0)	**0.610**	**<0.001**
	Neo/Adjuvant CT	0 (0.0)	**116 (92.1)**	6 (21.4)	7 (21.2)	45 (53.6)	2 (10.0)		
	Palliative CT	5 (55.6)	8 (6.3)	**21 (75.0)**	0 (0.0)	2 (2.4)	0 (0.0)		
	Neo/Adjuvant CRT	0 (0.0)	1 (0.8)	1 (3.6)	**25 (75.8)**	0 (0.0)	0 (0.0)		
	Active surveillance	0 (0.0)	1 (0.8)	0 (0.0)	1 (3.0)	37 (44.0)	0 (0.0)		
	Additional tests	0 (0.0)	0 (0.0)	0 (0.0)	0 (0.0)	0 (0.0)	**18 (90.0)**		

Bold text indicates statistically significant values. Abbreviations: LLM, large language model; CT, chemotherapy; CRT, chemoradiotherapy; AS, active surveillance; IP, interventional procedures.

**Table 3:** Performance Metrics of Large Language Models (guideline-integrated general-purpose LLM vs. domain-specialized model operating without external retrieval) in Comparison with Multidisciplinary Tumor Board Decisions.

LLM	Accuracy (%)	F1 Score	Recall	Cohen’s Kappa	*p* Value
Gemini 2.5	85.0	0.79	0.82	0.792	**<0.001**
MedGemma T = 0	70.3	0.64	0.61	0.566	**<0.001**
MedGemma T = 1	73.5	0.71	0.66	0.610	**<0.001**

Bold text indicates statistically significant values. Abbreviations: LLM, large language model.

**Table 4:** Comparative Analysis of Concordance and Clinical Safety of Gemini and MedGemma Large Language Models.

		Gemini 2.5 *n* (%)	MedGemma T = 0.0 *n* (%)	MedGemma T = 1.0 *n* (%)	*p* ^1^	*p* ^2^	*p* ^3^
**Concordance**	**Fully concordant**	245 (81.7)	171 (57.0)	187 (62.3)	<0.001 *	<0.001 *	0.038 *
	**Partially concordant**	23 (7.7)	64 (21.3)	54 (18.0)			
	**Discordant**	32 (10.7)	65 (21.7)	59 (19.7)			
**Safety**	**Safe**	251 (83.7)	187 (62.3)	188 (62.7)	<0.001 *	<0.001 *	0.567
	**Potentially risky**	42 (14.0)	76 (25.3)	68 (22.7)			
	**Harmful**	7 (2.3)	37 (12.3)	44 (14.7)			

Note: Comparisons were performed using the Marginal Homogeneity (Stuart–Maxwell) test, which is appropriate for paired multicategory data. *p*^1^ denotes the comparison between Gemini 2.5 and MedGemma (T = 0.0); *p*^2^ denotes the comparison between Gemini 2.5 and MedGemma (T = 1.0); and *p*^3^ denotes the comparison between MedGemma (T = 0.0) and MedGemma (T = 1.0). * *p* value < 0.05 was considered statistically significant. Bold text indicates statistically significant values.

**Table 5:** Comparative Analysis of Concordance Distributions Between Large Language Models Across Demographic and Clinical Variables.

		Gemini 2.5				MedGemma T = 0.0				MedGemma T = 1.0			
Variable	Group	FC (%)	PC (%)	D (%)	*p*	FC (%)	PC (%)	D (%)	*p*	FC (%)	PC (%)	D (%)	*p*
**Sex**	**Male** **Female**	—	—	—	0.390	—	—	—	0.179	—	—	—	0.675
**Age**	**<65** **65–74** **≤75**	—	—	—	0.380	—	—	—	0.202	—	—	—	0.214
**TNM stage**	**I**	12.7	0.0	3.1	**0.033**	13.5	0.0	13.8	**<0.001**	13.4	0.0	11.9	**<0.001**
	**II**	33.5	21.7	37.5		22.2	43.8	50.8		23.0	44.4	54.2	
	**III**	39.2	52.2	28.1		47.4	43.8	12.3		47.6	37.0	13.6	
	**IV**	14.7	26.1	31.3		17.0	12.5	23.1		16.0	8.5	20.3	
**Comorb.** **index**	**0** **1–2** **≥3**	—	—	—	0.485	—	—	—	0.545	—	—	15.3	0.246
**ECOG PS**	**0–1**	—	—	—	0.088	86.5	76.6	72.3	**0.023**	85.6	77.8	71.2	**0.036**
	**≥2**	—	—	—		13.5	23.4	27.7		14.4	22.2	28.8	
**Localization**	**Colon Rectum**	—	—	—	0.522	—	—	—	0.814	—	—	—	0.393
**MSI**	**Stabil**	—	—	—	0.246	60.2	73.4	67.7	**<0.001**	61.0	83.3	59.3	**<0.001**
	**İnstabil**	—	—	—		3.5	10.9	13.8		3.7	5.6	20.3	
	**Unknown**	—	—	—		36.3	15.6	18.5		35.3	11.1	20.3	
**Treatment**	**Adjuvant**	74.7	65.2	59.4	**<0.001**	68.4	79.7	75.4	**0.010**	70.6	70.4	79.7	**0.008**
**setting**	**Neoadjuvant**	9.0	8.7	6.3		12.9	3.1	3.1		12.3	3.7	1.7	
	**Metastatic**	13.1	21.7	9.4		15.2	12.5	9.2		13.4	20.4	6.8	
	**Recurrence**	3.3	4.3	25.0		3.5	4.7	12.3		3.7	5.6	11.9	
**MTB** **treatment** **modality**	**Surgery/IP**	1.2	0.0	18.8	**<0.001**	0.6	1.6	10.8	**<0.001**	1.1	1.9	10.2	**<0.001**
	**Neo/Adj CT**	41.2	47.8	43.8		46.2	64.1	9.2		45.5	63.0	11.9	
	**Palliative CT**	9.4	13.0	6.3		9.4	9.4	9.2		8.6	16.7	5.1	
	**Neo/Adj CRT**	9.8	26.1	9.4		12.9	17.2	0.0		12.8	14.8	1.7	
	**Active surveillance**	31.0	4.3	21.9		19.9	6.3	70.8		21.4	3.7	71.2	
	**Additional tests**	7.3	8.7	0.0		11.1	1.6	0.0		10.7	0.0	0.0	

Note: Differences between groups were analyzed using the chi-square test. Bonferroni correction was applied for multiple comparisons. *p* value < 0.05 was considered statistically significant. Abbreviations: FC, full concordance; PC, partial concordance; D, discordance; TNM, Tumor–Node–Metastasis; ECOG, Eastern Cooperative Oncology Group; CT, chemotherapy; CRT, chemoradiotherapy; IP, interventional procedures. Bold text indicates statistically significant values.

**Table 6:** Comparative Analysis of Clinical Safety of Large Language Models Across Demographic and Clinical Variables.

		Gemini 2.5				MedGemma T = 0.0				MedGemma T = 1.0			
Variables	Group	Safe (%)	Pot. Risky (%)	Harm. (%)	*p*	Safe (%)	Pot. Risky (%)	Harm. (%)	*p*	Safe (%)	Pot. Risky (%)	Harm. (%)	*p*
**Sex**	**Female**	—	—	—	0.192	—	—	—	0.335	—	—	—	0.775
	**Male**	—	—	—		—	—	—		—	—	—	
**Age**	**<65**	—	—	—	0.279	33.7	19.7	21.6	**0.019**	32.4	27.9	13.6	0.099
	**65–74**	—	—	—		35.8	28.9	35.1		34.0	33.8	34.1	
	**≥75**	—	—	—		30.5	51.3	43.2		33.5	38.2	52.3	
**TNM stage**	**I**	12.4	2.4	0.0	**0.024**	12.3	0.0	24.3	**<0.001**	13.3	0.0	15.9	**<0.001**
	**II**	34.3	31.0	0.0		27.3	46.1	35.1		23.4	50.0	47.7	
	**III**	38.6	35.7	71.4		44.9	28.9	29.7		47.3	26.5	22.7	
	**IV**	14.7	31.0	28.6		15.5	25.0	10.8		16.0	23.5	13.6	
**Comorb.** **index**	**0**	—	—	—	0.359	—	—	—	0.698	—	—	—	0.18
	**1–2**	—	—	—		—	—	—		—	—	—	
	**≥3**	—	—	—		—	—	—		—	—	—	
**ECOG PS**	**0–1**	83.3	73.8	57.1	0.087	86.6	75.0	67.6	**0.006**	86.2	79.4	63.6	**0.002**
	**≥2**	16.7	26.2	42.9		13.4	25.0	32.4		13.8	20.6	36.4	
**Localization**	**Colon**	—	—	—	0.832	—	—	—	0.653	—	—	—	0.358
	**Rectum**	—	—	—		—	—	—		—	—	—	
**MSI**	**Stable**	—	—	—	0.122	62.0	76.3	54.1	**<0.001**	61.2	85.3	47.7	**<0.001**
	**Unstable**	—	—	—		4.3	6.6	24.3		3.7	1.5	31.8	
	**Unknown**	—	—	—		33.7	17.1	21.6		35.1	13.2	20.5	
**Treatment setting**	**Adjuvant**	73.7	69.0	42.9	**0.005**	71.1	69.7	83.8	**0.026**	70.7	69.1	84.1	**0.023**
	**Neoadjuvant**	9.6	2.4	14.3		11.8	3.9	2.7		12.2	2.9	2.3	
	**Metastatic**	12.7	11.9	42.9		13.9	14.5	8.1		13.3	17.6	6.8	
	**Nüks**	4.0	16.7	0.0		3.2	11.8	5.4		3.7	10.3	6.8	
**MTB** **treatment modality**	**Surgery/IP**	1.2	14.3	0.0	**<0.001**	0.5	7.9	5.4	**<0.001**	1.1	5.9	6.8	**<0.001**
	**Neo/Adj CT**	41.4	45.2	42.9		49.7	36.8	13.5		46.3	50.0	11.4	
	**Palliative CT**	9.2	9.5	14.3		8.6	13.2	5.4		8.5	14.7	4.5	
	**Neo/Adj CRT**	10.4	16.7	0.0		11.8	14.5	0.0		12.8	11.8	2.3	
	**AS**	30.7	9.5	42.9		19.3	26.3	75.7		20.7	17.6	75.0	
	**Additional tests**	7.2	4.8	0.0		10.2	1.3	0.0		10.6	0.0	0.0	

Note: Differences between groups were analyzed using the chi-square test. Bonferroni correction was applied for multiple comparisons. *p* value < 0.05 was considered statistically significant. Abbreviations: TNM, Tumor–Node–Metastasis; ECOG, Eastern Cooperative Oncology Group; MSI, microsatellite instability status; CT, chemotherapy; CRT, chemoradiotherapy; AS, active surveillance; IP, interventional procedures. Bold text indicates statistically significant values.

## Data Availability

The data presented in this study are available on reasonable request from the corresponding author. The data are not publicly available due to institutional privacy regulations and patient confidentiality.

## Supplementary Materials

The following supporting information can be downloaded at: https://www.mdpi.com/article/10.3390/curroncol33060309/s1, Table S1: Model-specific prompt formulation for Gemini 2.5; Table S2: Model-specific prompt formulation for MedGemma 27B.

## Abbreviations

The following abbreviations are used in this manuscript:

AI	Artificial Intelligence
AS	Active Surveillance
CDSS	Clinical Decision Support System
CI	Confidence Interval
CRC	Colorectal Cancer
CRT	Chemoradiotherapy
CT	Chemotherapy
ECOG	Eastern Cooperative Oncology Group
F1 score	Harmonic Mean of Precision and Recall
HIPAA	Health Insurance Portability and Accountability Act
IP	Interventional Procedures
IRB	Institutional Review Board
KVKK	Personal Data Protection Law of Türkiye
LC-ICL	Long-Context In-Context Learning
LLM	Large Language Model
MSI	Microsatellite Instability
MTB	Multidisciplinary Tumor Board
NCI	National Cancer Institute
PDQ	Physician Data Query
RAG	Retrieval-Augmented Generation
TNM	Tumor–Node–Metastasis
